# Monoclinic polymorph of *trans*-tetra­aquabis­[(4-pyridylsulfanyl)­acetato-*κN*]cobalt(II)

**DOI:** 10.1107/S1600536808023593

**Published:** 2008-07-31

**Authors:** Dušan Mikloš, Jozef Miklovič, Jan Moncol, Peter Segľa, Marian Koman

**Affiliations:** a Department of Inorganic Chemistry, Slovak, Technical University, Radlinského 9, SK-812 37, Bratislava, Slovakia; b Department of Chemistry, Faculty of Natural Science, University of St. Cyril and Methodius, SK-91701 Trnava, Slovakia

## Abstract

The crystal structure of the title compound, [Co(C_7_H_6_NO_2_S)_2_(H_2_O)_4_], is a polymorph of the structure first reported by Du, Zhao & Wang [(2004). *Dalton Trans*, pp. 2065–2072]. The asymmetric unit of the title compound contains one half-mol­ecule; the Co^II^ atom lies on an inversion centre in a distorted octa­hedral geometry coordinated by two N atoms of the pyridine rings of the 4-pyridylthio­acetate anions and four O atoms of water mol­ecules. In the crystal structure, inter­molecular O—H⋯O hydrogen bonds link the mol­ecules, forming a three-dimensional network.

## Related literature

For related literature, see: Bernstein *et al.* (1995 ▶); Chiang *et al.* (1993 ▶); Du *et al.* (2004 ▶); Du & Li (2006 ▶); Kondo *et al.* (2002 ▶); For related structures, see: Fang *et al.* (2004 ▶); Zhang *et al.* (2004 ▶).
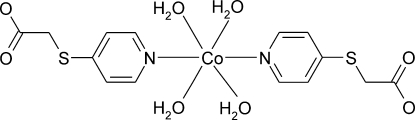


## Experimental

### 

#### Crystal data

[Co(C_7_H_6_NO_2_S)_2_(H_2_O)_4_]
*M* *_r_* = 467.37
Monoclinic, 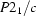

*a* = 12.173 (1) Å
*b* = 10.479 (1) Å
*c* = 7.523 (2) Å
β = 106.78 (3)°
*V* = 918.8 (3) Å^3^
*Z* = 2
Mo *K*α radiation
μ = 1.21 mm^−1^
*T* = 293 (2) K
0.45 × 0.40 × 0.30 mm

#### Data collection

Siemens P4 diffractometer
Absorption correction: ψ scan (*XEMP*; Siemens, 1994 ▶) *T*_min_ = 0.608, *T*_max_ = 0.684
3491 measured reflections
2651 independent reflections
2283 reflections with *I* > 2σ(*I*)
*R*_int_ = 0.024
3 standard reflections every 97 reflections intensity decay: 2.0%

#### Refinement

*R*[*F* ^2^ > 2σ(*F* ^2^)] = 0.032
*wR*(*F* ^2^) = 0.102
*S* = 1.37
2651 reflections
125 parameters
H-atom parameters constrained
Δρ_max_ = 0.41 e Å^−3^
Δρ_min_ = −0.37 e Å^−3^

### 

Data collection: *XSCANS* (Siemens, 1994 ▶); cell refinement: *XSCANS*; data reduction: *XSCANS*; program(s) used to solve structure: *SHELXS97* (Sheldrick, 2008 ▶); program(s) used to refine structure: *SHELXL97* (Sheldrick, 2008 ▶); molecular graphics: *ORTEP-3* (Farrugia, 1997 ▶); software used to prepare material for publication: *enCIFer* (Allen *et al.* 2004 ▶).

## Supplementary Material

Crystal structure: contains datablocks global, I. DOI: 10.1107/S1600536808023593/ez2135sup1.cif

Structure factors: contains datablocks I. DOI: 10.1107/S1600536808023593/ez2135Isup2.hkl

Additional supplementary materials:  crystallographic information; 3D view; checkCIF report

## Figures and Tables

**Table 1:** Hydrogen-bond geometry (Å, °)

*D*—H⋯*A*	*D*—H	H⋯*A*	*D*⋯*A*	*D*—H⋯*A*
O1*W*—H1*W*⋯O1^i^	0.82	2.05	2.849 (2)	163
O1*W*—H2*W*⋯O1^ii^	0.82	1.95	2.757 (2)	167
O2*W*—H3*W*⋯O2^ii^	0.82	1.91	2.725 (2)	176
O2*W*—H4*W*⋯O2^iii^	0.82	1.95	2.743 (2)	163

Symmetry codes: (i) 
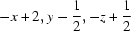
; (ii) 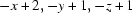; (iii) 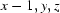.

**Fig. 1.**
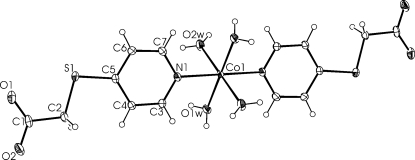
Molecular structure of the title compound, with atom-numbering scheme. Displacement ellipsoids are drawn at the 30% probability level.

**Fig. 2.**
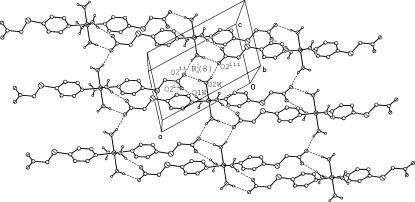
A layer of molecules of (I). Hydrogen bonds are shown as dashed lines [Symmetry codes: (ii) -x + 2, -y + 1, -z + 1; (iii) x - 1, y, z].

**Fig. 3.**
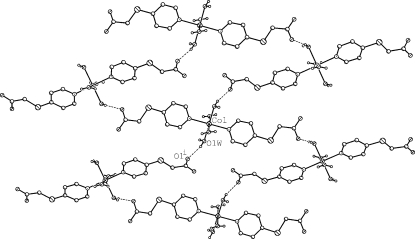
Hydrogen bonds between layers of molecules of (I) [Symmetry code: (i) -x + 2, y - 1/2, -z + 1/2].

## supplementary crystallographic information

### Comment

Several transition metal coordination polymers that contain bridging 4-pyridylthioacetate ligands have been reported recently (Chiang *et al.*, 1993; Du *et al.*, 2004; Du & Li, 2006; Kondo *et al.*, 2002). However, if the 4-pyridylthioacetate anions are coordinated only as terminal ligands there is a possibility that they may also be able to participate in a hydrogen-bonding network. As part of our efforts to investigate metal(II) complexes based on pyridyl-carboxylic acids, we report herein the crystal structure of the title compound, (I).

In the molecular structure of (I) (Fig. 1) the Co^II^ atom lies on an inversion centre and adopts a distorted octahedral coordination geometry with the two N atoms of the pyridine rings of the 4-pyridylthioacetate anions and the four O atoms of the water molecules, where the two symmetry related 4-pyridylthioacetate ligands are in *trans* positions.

The bond lengths and angles may be compared with the corresponding values in the triclinic polymorph [Co(C_7_H_6_NO_2_S)_2_(H_2_O)_4_] [(II); Du *et al.*, 2004]. In (II), the Co^II^ atom displays similar distorted octahedral coordination geometry, but the angle between the plane through the pyridine rings and that through the four water O atoms of 87.9° is closer to a right angle than the angle of 77.8° in (I). Correspondingly, the distance between the two planes of pyridine rings in (II) is shorter (0.22 Å) than that (0.80 Å) in (I). On the other hand, complex (I) is isostructural with [Cu(C_7_H_6_NO_2_S)_2_(H_2_O)_4_] [(III); Fang *et al.*, (2004)] and [Ni(C_7_H_6_NO_2_S)_2_(H_2_O)_4_] [(IV); Zhang *et al.*, (2004)].

In the crystal structure, intermolecular O–H···O hydrogen bonds (Table 1) link the molecules to form a three-dimensional network. The molecules of (I) lying in layers parallel to the *ac* plane are linked by O1W–H2W···O1^ii^; O2W–H3W···O2^ii^ and O2W–H4W···O2^iii^ [Symmetry codes: (ii) -*x* + 2, -*y* + 1, -*z* + 1; (iii) *x* - 1, *y*, *z*] hydrogen bonds (Fig. 2). The hydrogen bonds between two coordinated water molecules O2W and two carboxylate groups through only one carboxylate O atom (O2) of the carboxylate group create *R*_4_^2^(8) rings (Bernstein *et al.*, 1995). On the other hand, both O atoms of the two carboxylate groups and two coordinated water molecules create *R*_4_^4^(12) rings (Bernstein *et al.*, 1995) in the triclinic polymorph (II). The hydrogen bonds O1W–H1W···O1^i^ [Symmetry code: (i) -*x* + 2, *y* - 1/2, -*z* + 1/2] link the layers to form a 3-D hydrogen bonding network (Fig. 3).

### Experimental

Well shaped red crystals of (I) suitable for X-ray analysis were prepared in an H-tube. An aqueous solution of the sodium salt of 4-pyridylthioacetic acid, was placed in the first part of the H-tube, and an aqueous solution of Co(II) sulfate in the second part. Crystals formed after two weeks, whereafter they were separated and dried at room temperature (yield 70%). Anal. Calc. for C_14_H_20_CoN_2_O_8_S_2_: C, 35.98; H, 4.31; N, 5.99; S, 13.72; Co, 12.61. Found: C, 35.82; H, 4.41; N, 5.90; S, 13.59; Co, 12.75%. Selected IR data (cm^-1^): 1570 (*versus*,br) (ν_a_(COO^-^) + ν(C=N)), 1376 (*versus*) (ν_s_(COO^-^)), 430 (*m*) (γ(py), pyridine ring out-of-plane bending). Electronic data (cm^-1^): 21200, 20300, 9200br.

### Refinement

All H atoms of C–H (aromatic and methylene) were placed in calculated positions (0.93 and 0.97 Å, respectively); isotropic displaced parameters were fixed [*U*_iso_(H) = 1.2 *U*_iso_(C) of C atoms to which they were attached] using a riding model. The water H atoms were placed in calculated positions (O–H = 0.82 Å); isotropic displacement parameters were fixed [*U*_iso_(H) = 1.5*U*_iso_(O)) of O atoms to which they were attached].

### Crystal data

**Table:**

[Co(C_7_H_6_NO_2_S)_2_(H_2_O)_4_]	*F*_000_ = 482
*M**_r_* = 467.37	*D*_x_ = 1.689 Mg m^−^^3^
Monoclinic, *P*2_1_/*c*	Mo *K*α radiation λ = 0.71073 Å
Hall symbol: -P 2ybc	Cell parameters from 25 reflections
*a* = 12.173 (1) Å	θ = 1.7–7.9º
*b* = 10.479 (1) Å	µ = 1.21 mm^−^^1^
*c* = 7.523 (2) Å	*T* = 293 (2) K
β = 106.78 (3)º	Block, pink
*V* = 918.8 (3) Å^3^	0.45 × 0.40 × 0.30 mm
*Z* = 2	

### Data collection

**Table:**

Siemens P4 diffractometer	*R*_int_ = 0.024
Radiation source: fine-focus sealed tube	θ_max_ = 30.0º
Monochromator: graphite	θ_min_ = 1.8º
*T* = 293(2) K	*h* = −17→16
2θ/ω scans	*k* = −14→1
Absorption correction: ψ scan(XEMP; Siemens, 1994)	*l* = −1→10
*T*_min_ = 0.608, *T*_max_ = 0.684	3 standard reflections
3491 measured reflections	every 97 reflections
2651 independent reflections	intensity decay: 2.0%
2283 reflections with *I* > 2σ(*I*)	

### Refinement

**Table:**

Refinement on *F*^2^	Hydrogen site location: inferred from neighbouring sites
Least-squares matrix: full	H-atom parameters constrained
*R*[*F*^2^ > 2σ(*F*^2^)] = 0.032	*w* = 1/[σ^2^(*F*_o_^2^) + (0.0291*P*)^2^ + 0.3487*P*] where *P* = (*F*_o_^2^ + 2*F*_c_^2^)/3
*wR*(*F*^2^) = 0.102	(Δ/σ)_max_ = 0.001
*S* = 1.37	Δρ_max_ = 0.41 e Å^−^^3^
2651 reflections	Δρ_min_ = −0.37 e Å^−^^3^
125 parameters	Extinction correction: SHELXL, Fc^*^=kFc[1+0.001xFc^2^λ^3^/sin(2θ)]^-1/4^
Primary atom site location: structure-invariant direct methods	Extinction coefficient: 0.073 (6)
Secondary atom site location: difference Fourier map	

### Special details

**Table:**

Geometry. All e.s.d.'s (except the e.s.d. in the dihedral angle between two l.s. planes) are estimated using the full covariance matrix. The cell e.s.d.'s are taken into account individually in the estimation of e.s.d.'s in distances, angles and torsion angles; correlations between e.s.d.'s in cell parameters are only used when they are defined by crystal symmetry. An approximate (isotropic) treatment of cell e.s.d.'s is used for estimating e.s.d.'s involving l.s. planes.
Refinement. Refinement of *F*^2^ against ALL reflections. The weighted *R*-factor *wR* and goodness of fit *S* are based on *F*^2^, conventional *R*-factors *R* are based on *F*, with *F* set to zero for negative *F*^2^. The threshold expression of *F*^2^ > σ(*F*^2^) is used only for calculating *R*-factors(gt) *etc*. and is not relevant to the choice of reflections for refinement. *R*-factors based on *F*^2^ are statistically about twice as large as those based on *F*, and *R*- factors based on ALL data will be even larger.

### Fractional atomic coordinates and isotropic or equivalent isotropic displacement parameters (Å^2^)

**Table:**

	*x*	*y*	*z*	*U*_iso_*/*U*_eq_	
Co1	0.5000	0.5000	0.0000	0.01988 (13)	
S1	1.03489 (4)	0.69603 (5)	0.41990 (8)	0.03026 (16)	
O1	1.27843 (14)	0.69566 (16)	0.5784 (2)	0.0379 (4)	
O2	1.32163 (14)	0.52026 (19)	0.4432 (3)	0.0413 (4)	
O1W	0.56051 (12)	0.31434 (14)	0.0783 (2)	0.0303 (3)	
H1W	0.5952	0.2790	0.0135	0.046*	
H2W	0.5996	0.3114	0.1871	0.046*	
O2W	0.48936 (14)	0.5278 (2)	0.2660 (2)	0.0386 (4)	
H3W	0.5470	0.5169	0.3536	0.058*	
H4W	0.4300	0.5267	0.2967	0.058*	
N1	0.67362 (13)	0.57244 (16)	0.0968 (2)	0.0246 (3)	
C1	1.25327 (16)	0.6025 (2)	0.4709 (3)	0.0294 (4)	
C2	1.12871 (16)	0.5827 (2)	0.3577 (3)	0.0296 (4)	
H2A	1.1051	0.4969	0.3782	0.036*	
H2B	1.1223	0.5915	0.2267	0.036*	
C3	0.76428 (16)	0.50244 (19)	0.0894 (3)	0.0249 (4)	
H3	0.7508	0.4274	0.0205	0.030*	
C4	0.87692 (16)	0.5353 (2)	0.1785 (3)	0.0257 (4)	
H4	0.9369	0.4834	0.1686	0.031*	
C5	0.89935 (15)	0.64675 (19)	0.2828 (3)	0.0228 (4)	
C6	0.80572 (17)	0.7236 (2)	0.2835 (3)	0.0305 (4)	
H6	0.8171	0.8014	0.3458	0.037*	
C7	0.69607 (17)	0.6831 (2)	0.1909 (3)	0.0314 (5)	
H7	0.6346	0.7350	0.1938	0.038*	

### Atomic displacement parameters (Å^2^)

**Table:**

	*U*^11^	*U*^22^	*U*^33^	*U*^12^	*U*^13^	*U*^23^
Co1	0.01396 (18)	0.0244 (2)	0.02025 (19)	0.00132 (12)	0.00333 (12)	−0.00054 (13)
S1	0.0187 (2)	0.0350 (3)	0.0329 (3)	−0.00349 (18)	0.00085 (18)	−0.0080 (2)
O1	0.0261 (7)	0.0401 (9)	0.0392 (9)	−0.0061 (7)	−0.0036 (6)	0.0038 (7)
O2	0.0210 (7)	0.0621 (12)	0.0403 (9)	0.0061 (7)	0.0078 (6)	0.0027 (8)
O1W	0.0248 (7)	0.0294 (7)	0.0337 (8)	0.0049 (6)	0.0034 (6)	0.0022 (6)
O2W	0.0234 (7)	0.0702 (12)	0.0225 (7)	0.0067 (7)	0.0068 (6)	−0.0021 (7)
N1	0.0169 (7)	0.0273 (8)	0.0281 (8)	0.0001 (6)	0.0041 (6)	−0.0015 (6)
C1	0.0168 (8)	0.0434 (12)	0.0263 (9)	−0.0039 (8)	0.0034 (7)	0.0109 (9)
C2	0.0172 (8)	0.0392 (11)	0.0297 (10)	0.0010 (7)	0.0026 (7)	−0.0015 (8)
C3	0.0189 (8)	0.0261 (9)	0.0287 (9)	−0.0022 (7)	0.0049 (7)	−0.0042 (8)
C4	0.0169 (8)	0.0272 (9)	0.0317 (10)	0.0009 (7)	0.0050 (7)	−0.0023 (8)
C5	0.0173 (8)	0.0266 (9)	0.0230 (8)	−0.0013 (6)	0.0033 (6)	0.0005 (7)
C6	0.0226 (9)	0.0286 (10)	0.0382 (11)	−0.0003 (7)	0.0053 (8)	−0.0108 (8)
C7	0.0197 (9)	0.0307 (10)	0.0416 (12)	0.0037 (7)	0.0053 (8)	−0.0067 (9)

### Geometric parameters (Å, °)

**Table:**

Co1—O2W^i^	2.0632 (16)	N1—C3	1.340 (2)
Co1—O2W	2.0632 (16)	N1—C7	1.345 (3)
Co1—O1W^i^	2.1034 (15)	C1—C2	1.524 (3)
Co1—O1W	2.1034 (15)	C2—H2A	0.9700
Co1—N1^i^	2.1644 (16)	C2—H2B	0.9700
Co1—N1	2.1644 (16)	C3—C4	1.385 (3)
S1—C5	1.7523 (19)	C3—H3	0.9300
S1—C2	1.800 (2)	C4—C5	1.389 (3)
O1—C1	1.248 (3)	C4—H4	0.9300
O2—C1	1.257 (3)	C5—C6	1.397 (3)
O1W—H1W	0.8200	C6—C7	1.382 (3)
O1W—H2W	0.8200	C6—H6	0.9300
O2W—H3W	0.8200	C7—H7	0.9300
O2W—H4W	0.8200		
			
O2W^i^—Co1—O2W	180.0	O1—C1—O2	126.42 (19)
O2W^i^—Co1—O1W^i^	88.52 (7)	O1—C1—C2	119.1 (2)
O2W—Co1—O1W^i^	91.48 (7)	O2—C1—C2	114.4 (2)
O2W^i^—Co1—O1W	91.48 (7)	C1—C2—S1	111.63 (16)
O2W—Co1—O1W	88.52 (7)	C1—C2—H2A	109.3
O1W^i^—Co1—O1W	180.0	S1—C2—H2A	109.3
O2W^i^—Co1—N1^i^	87.28 (7)	C1—C2—H2B	109.3
O2W—Co1—N1^i^	92.72 (7)	S1—C2—H2B	109.3
O1W^i^—Co1—N1^i^	90.07 (6)	H2A—C2—H2B	108.0
O1W—Co1—N1^i^	89.93 (6)	N1—C3—C4	123.78 (18)
O2W^i^—Co1—N1	92.72 (7)	N1—C3—H3	118.1
O2W—Co1—N1	87.28 (7)	C4—C3—H3	118.1
O1W^i^—Co1—N1	89.93 (6)	C3—C4—C5	119.23 (18)
O1W—Co1—N1	90.07 (6)	C3—C4—H4	120.4
N1^i^—Co1—N1	180.0	C5—C4—H4	120.4
C5—S1—C2	102.29 (10)	C4—C5—C6	117.35 (17)
Co1—O1W—H1W	116.8	C4—C5—S1	125.34 (15)
Co1—O1W—H2W	111.7	C6—C5—S1	117.27 (15)
H1W—O1W—H2W	109.0	C7—C6—C5	119.40 (19)
Co1—O2W—H3W	118.7	C7—C6—H6	120.3
Co1—O2W—H4W	125.3	C5—C6—H6	120.3
H3W—O2W—H4W	113.0	N1—C7—C6	123.38 (18)
C3—N1—C7	116.71 (16)	N1—C7—H7	118.3
C3—N1—Co1	122.00 (13)	C6—C7—H7	118.3
C7—N1—Co1	120.65 (13)		
			
O2W^i^—Co1—N1—C3	−59.79 (17)	Co1—N1—C3—C4	−168.13 (16)
O2W—Co1—N1—C3	120.21 (17)	N1—C3—C4—C5	0.2 (3)
O1W^i^—Co1—N1—C3	−148.31 (17)	C3—C4—C5—C6	−3.3 (3)
O1W—Co1—N1—C3	31.69 (17)	C3—C4—C5—S1	174.30 (16)
O2W^i^—Co1—N1—C7	129.63 (17)	C2—S1—C5—C4	8.6 (2)
O2W—Co1—N1—C7	−50.37 (17)	C2—S1—C5—C6	−173.75 (17)
O1W^i^—Co1—N1—C7	41.12 (17)	C4—C5—C6—C7	3.5 (3)
O1W—Co1—N1—C7	−138.88 (17)	S1—C5—C6—C7	−174.29 (18)
O1—C1—C2—S1	−5.4 (3)	C3—N1—C7—C6	−2.6 (3)
O2—C1—C2—S1	175.01 (16)	Co1—N1—C7—C6	168.50 (19)
C5—S1—C2—C1	−176.55 (15)	C5—C6—C7—N1	−0.6 (4)
C7—N1—C3—C4	2.8 (3)		

Symmetry codes: (i) −*x*+1, −*y*+1, −*z*.

### Hydrogen-bond geometry (Å, °)

**Table:**

*D*—H···*A*	*D*—H	H···*A*	*D*···*A*	*D*—H···*A*
O1W—H1W···O1^ii^	0.82	2.05	2.849 (2)	163
O1W—H2W···O1^iii^	0.82	1.95	2.757 (2)	167
O2W—H3W···O2^iii^	0.82	1.91	2.725 (2)	176
O2W—H4W···O2^iv^	0.82	1.95	2.743 (2)	163

Symmetry codes: (ii) −*x*+2, *y*−1/2, −*z*+1/2; (iii) −*x*+2, −*y*+1, −*z*+1; (iv) *x*−1, *y*, *z*.
